# Automated and scalable expansion of human liver organoids for translational applications

**DOI:** 10.1186/s12967-026-08169-z

**Published:** 2026-04-24

**Authors:** Marjolein J.M. ten Dam, Juda-El S. Jno Baptiste - Sam, Rachelle S. Schwanen, Lisa van Uden, Monique M.A. Verstegen, Luc J.W. van der Laan, Sabine A. Fuchs, Ruud Das, Bart Spee

**Affiliations:** 1 Department Biomedical Engineering, Eindhoven Technical University, Eindhoven, The Netherlands; 2 Scinus Cell Expansion Netherlands B.V., Zeist, The Netherlands; 3 Department of Clinical Sciences, Faculty of Veterinary Medicine, Utrecht University, Utrecht, The Netherlands; 4 Department of Surgery, Erasmus MC Transplant Institute, Erasmus University Medical Center, Rotterdam, The Netherlands; 5 Department of Metabolic Diseases, Wilhelmina Children’s Hospital, University Medical Center Utrecht, Utrecht, The Netherlands

**Keywords:** Liver organoids, Automated bioreactor, Large-scale expansion, Suspension culture, Phenotypic stability, Hepatic differentiation, Regenerative medicine, Cell therapy, Disease modeling, Translation applications

## Abstract

**Background:**

Human tissue-derived organoids hold strong potential for personalized medicine and cell therapy, but this requires large cell quantities. Conventional organoid culture systems remain labor-intensive, are difficult to scale, and lack process control. Here, we present a novel strategy using an automated bioreactor platform that enables large-scale expansion of human liver organoids.

**Methods:**

Human liver organoids were expanded for 14 days in a single bioreactor suspension culture bag and compared with spinner flasks and static dome cultures. Cell yield, viability, fold expansion, morphology, and phenotypic markers (LGR5, E-cadherin, Vimentin, Ki67) were assessed. The system’s uninterrupted workflow enabled seamless transition to differentiation: using integrated perfusion, we performed a direct medium switch from expansion to hepatic differentiation without harvesting or disrupting the culture. Commitment to the hepatic lineage was evaluated by expression of ALB, CYP3A4, MRP2, and HNF4A.

**Results:**

By day 14, the bioreactor generated an average of 5.63 × 10^8^ (± 1.1 × 10^8^) viable cells, while spinner flasks reached 1.22 × 10^8^ (± 4.26 × 10^7^) cells, while static cultures yielded only 4.02 × 10^5^ (± 2.81 × 10^5^), making the bioreactor’s output ~ 1400 times greater than static cultures (*p* = 0.022) and nearly five times higher than spinner flasks. This substantial gain in absolute cell yield is a promising indicator for downstream translation. Organoids preserved phenotypic integrity and proliferative capacity as shown by sustained expression of LGR5, E-cadherin, and Ki67. Bioreactor-cultured organoids exhibited robust growth and intact cyst-like morphology with a large size, due to the absence of mechanical fragmentation and related cellular stress. As a proof-of-principle, bioreactor-grown organoids differentiated efficiently toward the hepatic lineage, as evidenced by a downregulated gene expression of LGR5 and Ki67, with elevated gene expression of *ALB*, *CYP3A4*, *MRP2*, and *HNF4A*, along with an upregulated secretion of Albumin.

**Conclusion:**

The system establishes a closed, monitored, and scalable upstream workflow for liver organoid expansion. This work represents a significant step toward organoid production for future cell therapy and regenerative medicine applications, while maintaining phenotypic stability and differentiation capacity.

**Supplementary Information:**

The online version contains supplementary material available at 10.1186/s12967-026-08169-z.

## Background

Liver transplantation remains the only curative treatment for end-stage liver failure, yet most patients never receive a new liver due to an overwhelming shortage of suitable donor organs [1, 2]. The increasing demand, combined with the unsuitability of most donated livers for transplantation, underscores the urgent need for alternative regenerative solutions [2, 3]. Adult stem cell-derived liver organoids have emerged as a promising method for generating large quantities of liver cells [4, 5]. These organoids not only expand rapidly in vitro but also closely mimic the in vivo structure and function of human liver tissue [6, 7]. In a recent review by our group, we highlighted the broad translational potential of liver organoids, particularly intrahepatic cholangiocyte organoids (ICOs), to alleviate the burden on the transplant waiting list, either by direct transplantation into patients to restore hepatic function or by regenerating bile ducts in damaged donor livers to improve transplant viability [8]. Among the different liver organoid types, ICOs uniquely combine broad applicability with robust expansion potential, making them a leading candidate not only for clinical upscaling but also for disease modeling and drug discovery applications [7, 9–11].

The number of cells required for transplantation is highly dependent on the disease severity, delivery method, and engraftment efficiency. For hepatocyte transplantation, cell doses of at least 1–2 × 10^8^ cells/kg body weight [12, 13] are required, translating to over 6 billion cells for an average adult. Even though these numbers appear significant, hepatic organoid (HOs) transplantation has already been shown to be successful. *Nie et al.,* showed that HOs could rescue acute liver failure in mice [14], and *Kruitwagen et al.,* reported presence of autologous corrected organoids two years following implantation in a COMMD1-deficient canine model [15]. In contrast, biliary regeneration requires a markedly lower cell dose: around 1 × 10^6^ cholangiocyte organoids/kg body weight was sufficient to restore bile duct function in both murine and ex vivo human liver models [16]. The optimal transplantation route for clinical applications will depend on the target cell type and disease context. To meet these therapeutic demands, scalable, reproducible, and Good Manufacturing Practice (GMP) compliant culture systems are required to deliver high cell yields with minimal manual intervention [17–20]. Yet, current organoid culture methods rely heavily on static droplet cultures embedded in laminin-rich matrices such as Matrigel, which are highly labor- and time-intensive, material-heavy, and rely solely on diffusion for nutrient and oxygen transport [4, 21, 22]. Flow in an agitation system can enhance the exchange of nutrients and oxygen by homogeneously mixing the culture medium, thereby boosting cellular metabolism and improving proliferation rates, as has already been demonstrated in spinner flasks [23, 24]. However, spinner flasks remain small-scale open systems that increase the risk of contamination, are limited to basic CO_2_ incubator gas control, lack online monitoring capabilities, and subject cells to high shear forces. Cells exposed to chronic environmental or desynchronization stress undergo profound transcriptional and metabolic adaptations, including reprogramming of circadian regulators and stress response pathways, which may similarly shape organoid adaptation to fluctuating culture conditions [25–27]. Automated cell expansion systems, such as bioreactors, not only offer continuous and precise process control but also reduces the time required for upscaling, decreases material costs and minimizes variability caused by human intervention [8, 28, 29].

In this study, we demonstrate liver organoid expansion in an automated bioreactor and compare its performance to standardized static droplet and spinner flasks cultures. Specifically, we tested whether an automated bioreactor enables high-yield ICO expansion while preserving their epithelial identity and differentiation capacity. Within just two weeks, the platform achieved robust organoid proliferation while preserving the proliferative epithelial stem cell phenotype, yielding an average of 5.63 × 10^8^ cells from a single culture bag (*n* = 4). Following expansion, hepatic differentiation was initiated in the same bioreactor run through a seamless medium switch, serving as a proof-of-principle for downstream applications. Together, these findings establish a high-yield, closed-system workflow that integrates single-use culture bags, real-time monitoring, and process automation, positioning bioreactor-based organoid expansion as a promising closed, monitored manufacturing workflow for therapeutic, disease-modeling, and drug-discovery applications.

## Methods

### Human intrahepatic cholangiocyte organoids (ICOs) establishment

Liver tissue was acquired from four different donors (3 DBD, 1 DCD), in accordance with protocols approved by the institutional ethics committee (Suppl., Table S1), after which it was minced into smaller pieces using a scalpel while submerged in cold 1% DMEM (DMEM Glutamax (Gibco) with 1% (v/v) P/S (Gibco)). Subsequently, the tissue was dissociated by immersing it in a 15 mL collagenase-dispase enzyme solution containing 125 µg/mL collagenase II (Gibco), 125 µg/mL dispase (Gibco), and 100 µg/mL DNase I (Roche) and incubated in a 37 °C water bath until the tissue is degraded. Every 30 min, the tissue was mechanically disrupted and allowed to sink to the bottom of the tube before the supernatant was collected and transferred to a 15 mL tube on ice. Then, 15 ml fresh dissociation solution was again added to the settled tissue, and the dissociation process was repeated until all tissue was digested. All collected supernatants were centrifuged at 450 ×*g* for 5 min at room temperature. Pellets were resuspended in 1 mL 1% DMEM and pooled. Advanced DMEM (Gibco) was added to the suspension until a total volume of 13 mL was reached, after which the suspension was centrifuged at 500 ×*g* for 5 min at 4 °C. Supernatant was removed, and cells were distributed into Matrigel droplets for 1–8 wells depending on the cell pellet on prewarmed culture plates. Cells and Matrigel were incubated at 37 °C in a humidified atmosphere of 5% CO_2_ in air for 15 min to allow Matrigel polymerization. Finally, for the first 3 days, ICO expansion medium (EM; Suppl., Table S4) supplemented with 30% v/v Wnt3a-conditioned medium, 10 µM y-27632, and 25 ng/ml noggin was added to each well. Organoid formation was monitored over the next days, and media was refreshed every 2–3 days. After establishment, ICOs were expanded in EM at 37 °C in a humidified atmosphere of 5% CO_2_ in air. Medium was refreshed every 2–3 days. Organoids were passaged via mechanical fragmentation and 1x TrypLE (Gibco, 12604013) incubation at 37 °C at a 90% confluency for a 1:4 average split ratio.

### ICO expansion in conventional static vs. dynamic culture systems

For each comparative experiment, static droplet, spinner flask, and bioreactor cultures were initiated in parallel from the same donor-derived ICO line, passage number, and pre-expansion batch, harvested at the same timepoint prior to seeding. Cell counts were obtained from the same dissociated organoid suspension, which was divided across the three culture modalities. Static droplet and spinner flask cultures were performed using standardized comparator protocols, whereas bioreactor cultures used platform-specific optimized operating conditions. Cells were counted and seeded according to the optimized densities per modality (Suppl., Table S3). For static cultures, 3 × 10^3^ cells were seeded in 100 µL Matrigel droplets per well in a 12-well plate and statically incubated for 14 days. For spinner flask cultures, 3.3 × 10^5^ cells were seeded in 22 mL (including 10% (v/v) Matrigel) in a 125-mL spinner flask (Corning, 3152). The stirring speed was set to 85 rpm. In the bioreactor suspension bags (Scinus Cell Expansion Netherlands, SUS-05-05) cells were seeded at the same density as spinner flask cultures, resulting in 1.5 × 10^6^ cells in 100 mL EM (including 5% (v/v) Matrigel). The rocker setpoint was set to continuously mix the culture at 250°/s with an angle of 90° (Table S2 for more parameter settings). In all cultures, ICOs were expanded for 14 days at 37 °C. Static and spinner cultures were incubated in a humidified atmosphere of 5% CO_2_ in air, while automated bioreactor cultures were maintained with a dissolved oxygen level (DO_2_) of 75% and pH of 7.3. Medium was refreshed every 2–3 days for static droplet cultures. However, for both dynamic culture systems, fresh EM (incl. 5% or 10% (v/v) Matrigel) was added every 2–3 days, thereby increasing its total volume each time (Suppl., Table S6).

### ICO differentiation in conventional static vs. dynamic culture systems

ICO differentiation towards the hepatic lineage was initiated with two days of priming in EM supplemented with 25 µg/mL BMP7, followed by 10 days of culture in hepatocyte differentiation medium (HDM) (Suppl., Table S5). For static droplet cultures, EM was simply removed and, after one washing step with Advanced DMEM/F12, replaced with an equal amount of HDM. Cultures were maintained at 37 °C in a humidified atmosphere of 5% CO_2_ in air, and media was refreshed every 2–3 days. To switch to HDM in spinner flasks, some additional steps are required. ICOs were allowed to sediment by temporarily stopping agitation, after which the majority of EM could be safely removed without removing the organoids. The remaining cell suspension was centrifuged at 250 ×*g* for 5 min at 4 °C and residual supernatant was removed. ICOs were washed with Advanced DMEM/F12 and centrifuged at 250 ×*g* for 5 min at 4 °C. After supernatant removal, the ICOs were gently resuspended in 64 mL HDM and reseeded in the same 125-mL spinner flask. Agitation speed was set to 85 rpm again. For bioreactor cultures, we made use of the 1 μm filter separating the two compartments in the suspension bag to aseptically remove EM from the top compartment while retaining both ICOs and Matrigel in the bottom compartment of the culture bag. Residual EM was diluted by washing with Advanced DMEM/F12, after which it was replaced by 400 mL HDM (excl. Matrigel). To both dynamic cultures, fresh HDM was added on top of the culture volume every 2–3 days (incl. 5% or 10% (v/v) Matrigel).

### The Osilaris™ system

The automated bioreactor is a closed system for the controlled expansion of suspension or adherent cell types with or without microcarriers [28]. Non-gas-permeable, single-use culture bags containing cells are housed on a rocking platform that allows for a homogenous culture environment through gentle wave-inducing mixing. Modifiable rocking/agitation regimes ensure uniform distribution of nutrients and oxygen throughout the bag according to the requirements of the culture type. Prior to the main experiments, an empirical parameter screen was performed to identify the lowest agitation regime that maintained uniform organoid distribution (i.e., minimal settling/aggregation during routine operation) while preserving organoid integrity, assessed by brightfield morphology and viability measurements at early time points. The selected operating regime used for all donor experiments was chosen based on these criteria. Hydrodynamic shear stress was not directly measured in this study and no CFD-based shear quantification was performed.

The suspension bag (Scinus Cell Expansion Netherlands, SUS-05-05) used here is divided into two compartments separated by a 1 μm pore-sized filter, designed to retain cells in an undisturbed environment while continuous perfusion is employed to maintain the pH and DO_2_ concentration throughout the culture period. Perfusion technology in the bioreactor is achieved by closed-loop medium circulation between the bag’s top compartment, through non-gas-permeable tubing, into gas-permeable tubing housed in the carboxygenator. User-defined DO_2_ and pH are then enabled by controlled diffusion of CO_2_, O_2_, and N_2_ gases in the carboxygenator and monitored using built-in sensor technology. Extracellular matrix (ECM) factors produced by cells can be maintained throughout the culture due to the volume expandable nature of the culture bags. At refreshing time points, users can expand the bag via the graphical user interface which controls the roller position on the rocker platform, thereby scaling the volume of the process in a single bag (0.1–1.2 L; Suppl. Table S6). Fresh medium can be added aseptically via weldable tubing ports of the bag using tube welders and sealers. Process automation from expansion all the way through to differentiation remains flexible as users may adjust process factors such as agitation regime and perfusion speed as required, without sacrificing the process. Additional to online process monitoring, expansion progress can be monitored offline by sterile culture sampling for cell counts and other analysis.

### Bioreactor parameters for large-scale expansion of liver organoids from single cells

Organoids were first enzymatically digested into single cells. Cell pellets were then resuspended in 100 mL cold expansion medium supplemented with 5% (v/v) Matrigel and aseptically seeded into each suspension bag at 1.5 × 10^4^ cells/mL cell density (Scinus Cell Expansion Netherlands, SUS-05-05). Over the period of 14 days, single cells formed varied sizes of translucent organoids with the help of an agitation regime of continuous rocking at 250°/s at an angle of 90°. The rocking speed was set to the lowest level that maintained homogeneous suspension and prevented organoid sedimentation, as assessed by visual inspection, while preserving intact, translucent organoid morphology and viability throughout culture. Sampling was performed at every refreshing time point, which was every 2–3 days. Cultures were refreshed aseptically by expanding the bag and adding extra culture medium. Experiments were performed in parallel. Further details on critical parameter settings for optimal organoid formation and expansion using the automated bioreactor are compiled in Supplemental Information Table S2.

### Quantification of cell numbers

Samples were counted with automated cell analyzers before seeding in different culture modalities. For bioreactor samples, the NucleoCounter^®^ NC-250™ automatic cell analyzer (ChemoMetec) was used, while the CytoSMART™ Exact FL (Axion Biosystems) was used for the spinner flask and static samples. Due to the different seeding volumes in each format, the actual cell counts differ on day 0. The seeding density, however, was kept consistent at 1.5 × 10^4^ cells/mL for organoids cultured both in the automated bioreactor and spinner flasks, while in static droplet cultures the seeding density was 3 × 10^4^ cells/mL. The (mean) viable cell counts over time per modality can be found in Supplemental Information Table S3.

### Metabolite measurements from culture medium

Glucose (GlucCell, CESCO bioengineering) and lactate (Lactate plus, Nova Biomedical) levels were measured over time in the culture medium from the automated bioreactor cultures following manufacturer’s protocol. In brief, a disposable test strip was inserted into the device, and a medium droplet was added to the end of the test strip. Within seconds, the data was obtained. The process was repeated at least two times to check the consistency of the measurement.

### RNA isolation and quantitative real-time PCR (qRT-PCR)

RNA was isolated using the RNeasy Mini Kit (Qiagen, 74106) according to the manufacturer’s instructions. For isolating RNA, organoids were collected and lysed with RLT buffer including 1% (v/v) β-mercaptoethanol. Lysates were transferred to a new tube and further processed according to the RNeasy Mini Kit protocol. Complementary DNA (cDNA) was synthesized using the iScript cDNA synthesis kit (Bio-Rad, Hercules, CA) as described by the manufacturer. Relative mRNA of the selected genes was measured by real-time PCR using validated primers (Table S7) with the SYBR Green method (Bio-Rad) using two replicates per gene. For normalization of the mRNA, the average of three reference genes (hypoxanthine-guanine phosphoribosyltransferase (HPRT), ribosomal protein L19 (RPL19), and Tyrosine 3-monooxygenase/tryptophan 5-monooxygenase activation protein zeta (YWHAZ) was used.

### Microscopy and wholemount immunofluorescent (IF) staining

Organoids were collected aseptically on sampling days and prepared according to previously published protocols [30]. In short, excess Matrigel was removed by incubating with cell recovery solution (Corning, 354253) for 30 min at 4 °C. Organoids were fixed with 4% (v/v) paraformaldehyde for 45 min at 4 °C and stored in 70% (v/v) ethanol until future use. Organoids were transferred to an IBIDI 24-well plate (IBIDI, 82426) and blocked for 15 min in organoid washing buffer (OWB; 1 g Bovine Serum Albumine and 100µL Triton X-100 in 100mL PBS). Samples were incubated with primary antibodies overnight at 4 °C (Table S8), and washed three times with OWB buffer for 2 h at 4 °C. Samples were incubated overnight with secondary antibodies at 4 °C on a horizontal shaker, and again washed in OWB buffer three times for 2 h at 4 °C. Finally, all OWB buffer was removed, and samples were incubated in fructose-glycerol clearing solution (2.5 M fructose, Sigma-Aldrich, F0127; and 60% (v/v) glycerol, Sigma-Aldrich, G9891) for 20 min at 37 °C. Samples were imaged using a confocal microscope (Leica TCS SP8). Immunofluorescence images were acquired using identical acquisition settings within each experiment and are shown as representative images; no quantitative fluorescence intensity was performed.

### Liver enzyme and protein secretion

Organoids were collected from the automated bioreactor, spinner flask, and static droplets in a 15 mL tube and centrifuged at 450 ×*g* for 5 min at 4 °C. Pellets were resuspended with undiluted Matrigel while on ice. Droplets were plated in a 12-wells plate and incubated for 10–30 min at 37 °C. 1 mL HDM was added to each well. After 24 h, the cell lysate was recollected and sent to Universitair Veterinair Diagnostisch Laboratorium (Utrecht, the Netherlands) to be analyzed. Values were normalized for cell numbers and provided in mg/L/million cells for Albumin and U/L/million cells for AST and ALT.

### Statistics

Statistical analysis was performed using GraphPad Prism (Version 10.4.1). Each donor-derived organoid line was considered one independent biological replicate (*n* = 4 donors). Where technical replicates were performed, values were averaged per donor prior to analysis. Because the same donor lines were evaluated across culture methods and timepoints, measurements were treated as repeated observations within donor. For the primary analyses of cell yield and gene expression, a two-way ANOVA was used to assess the effects of time (two timepoints) and technique (three methods), including their interaction. For fold increase in cell count, Tukey’s multiple-comparisons test was used to compare culture methods within each timepoint. For gene expression, Sidak’s multiple-comparisons test was used to compare timepoints within each culture method. For RT-qPCR, statistical testing was performed on [log-transformed expression values], while fold changes are shown for visualization. Sample size was based on the primary cell-yield endpoint: based on log-transformed pilot variability, *n* = 4 donors per method provided ≥ 80% power (α = 0.05, two-sided) to detect a 3-fold difference in cell yield (Δ = log(3) ≈ 1.10). Model residuals were assessed using quantile-quantile (Q-Q) plots and Shapiro-Wilk testing where appropriate. Statistical significance was defined as *p* < 0.05.

## Results

### Set-up of liver organoid expansion in the bioreactor

**Fig. 1:**
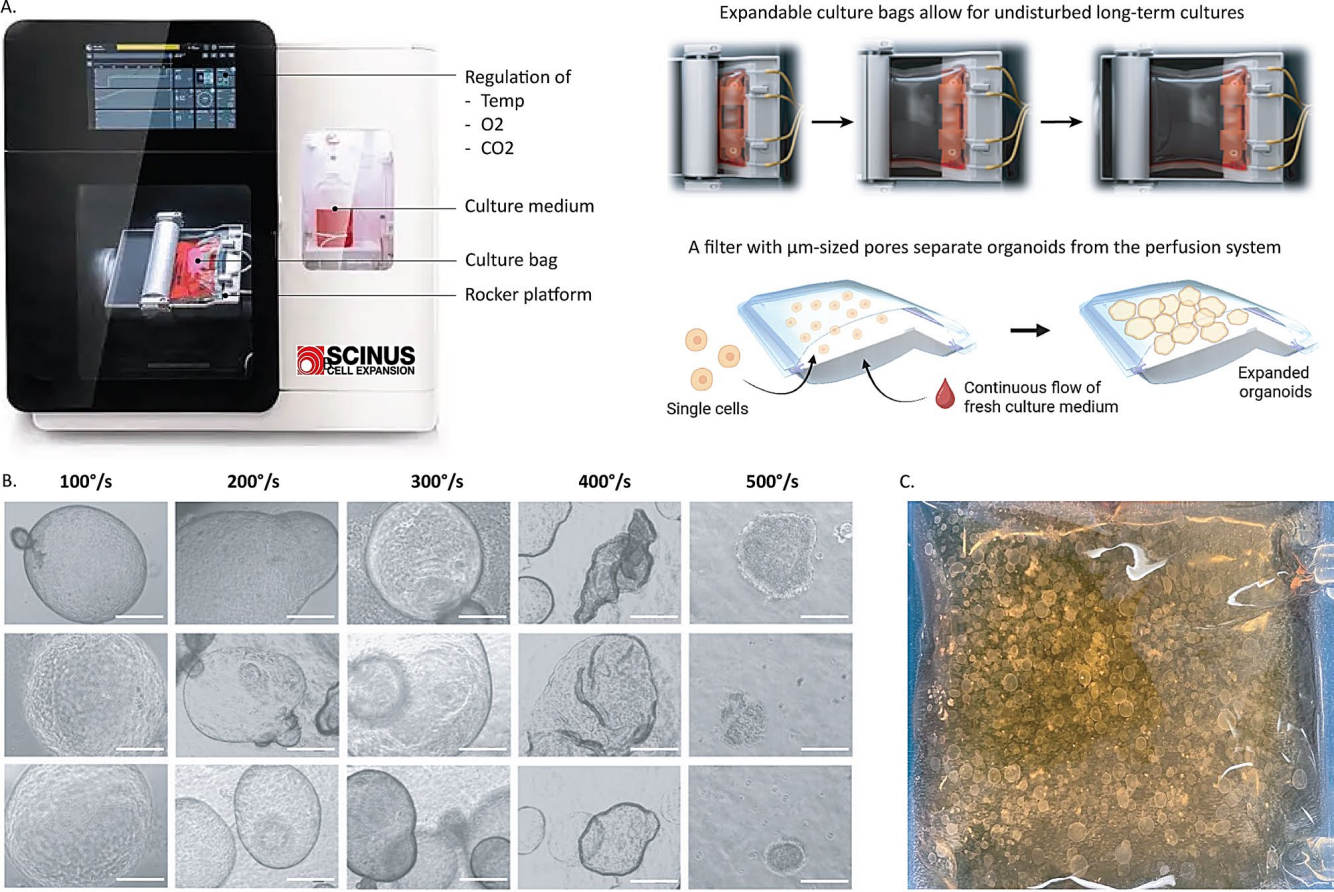
Organoids in automated bioreactor culture. **A**. Schematic representation of the automated bioreactor set-up, including the expandable suspension culture bags; **B**. Organoid morphology at different speeds, scale bar indicates 100 μm; **C**. Organoids in the small culture bag visible to the naked eye

The Osilaris*™* system comprises an instrument, single-use culture bags, and control software (Fig. 1A), supporting dynamic cultivation. Due to multiple degrees of freedom, it requires cell type-specific optimization, especially for the agitation regime. Five different agitation speeds (100–500°/s) were tested using whole, unfragmented ICOs (Suppl., Table S1, donor 1) in small-volume, gas-permeable suspension bags (Origen Biomedical, PL70-2G). Bags were incubated in a 5% CO_2_ controlled container fixed on the rocker platform. Continuous rocking above 300°/s led to disrupted morphology and reduced viability (Fig. 1B), while agitation below 200°/s or with a rocking angle < 90° resulted in poor organoid distribution. These observations were attributed to insufficient mixing, likely due to medium viscosity from Matrigel supplementation. Under optimal conditions, organoids reached large sizes visible to the naked eye (Fig. 1C), owing to the absence of mechanical disruption during culture. The platform introduces fresh culture medium, including Matrigel, via weldable tubing while continuously maintaining pH and DO_2_ setpoints through an integrated perfusion system. This design allows progressive culture expansion by increasing the bag volume, enabling uninterrupted growth during both the expansion and hepatic differentiation phases (Fig. 1A). Unlike traditional approaches requiring mechanical fragmentation to maintain culture density, organoids in the bioreactor remained physically untouched throughout the run. Based on these insights, the parameters (Suppl., Table S2) were set for subsequent large-scale experiments.

### Superior large-scale liver organoid expansion in the bioreactor

**Fig. 2:**
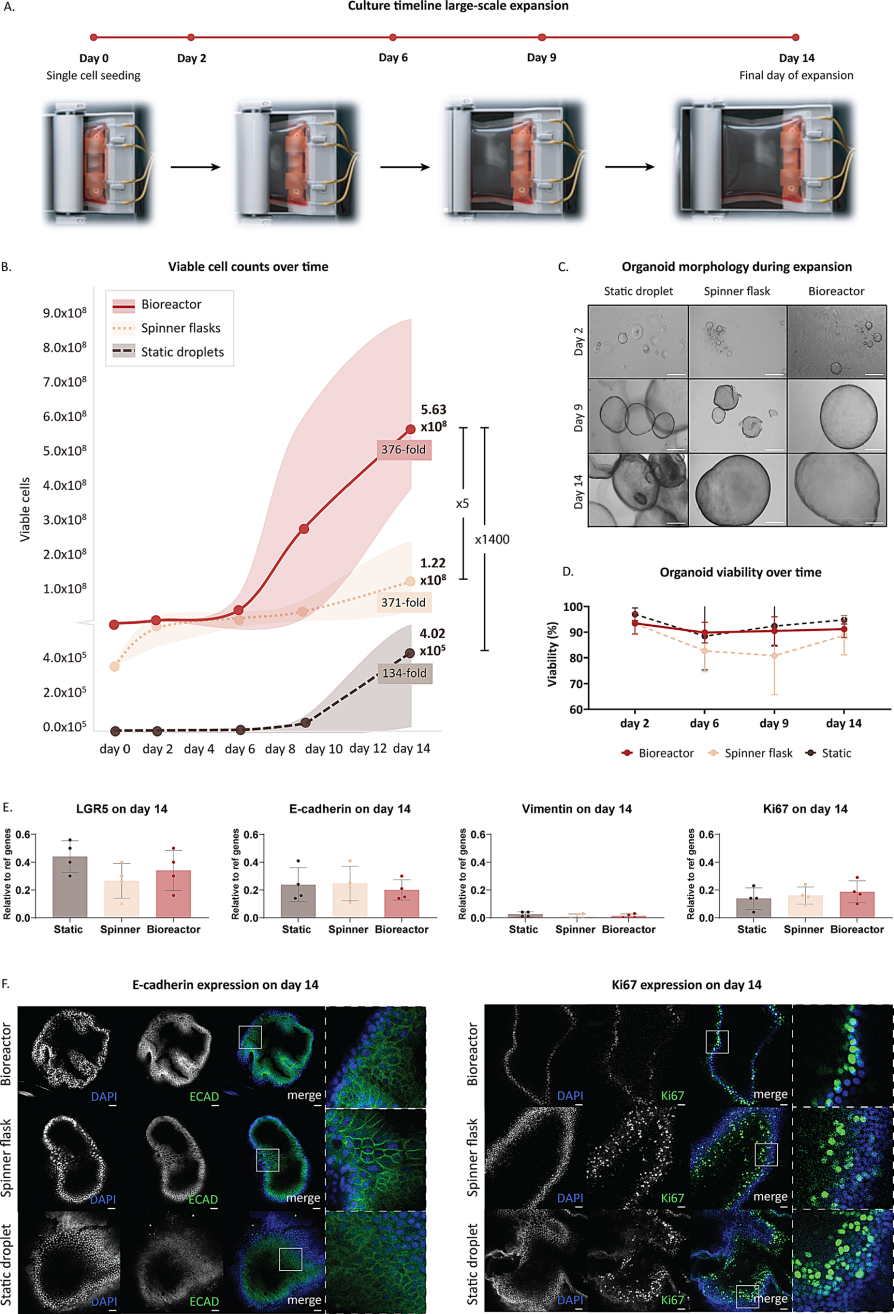
Superior large-scale liver organoid expansion in the bioreactor while maintaining an epithelial phenotype. **A**. Schematic timeline of the expansion culture protocol; **B**. Mean viable cell count from all four donors plotted per culture method (automated bioreactor, spinner flask, and static droplets) on days 2, 6, 9, and 14; **C**. Representative images show organoids growth over a 14-day expansion period, scale bar indicates 100 μm; **D**. Organoid viability over time for all modalities; **E**. Gene expression of stemness marker LGR5, epithelial marker E-cadherin, and proliferation marker Ki67, with absence of Vimentin at the end of the expansion phase; **F**. Immunostaining for E-cadherin indicates a sustained epithelial nature of liver organoids, while Ki67 indicates a sustained proliferative capacity of liver organoids after 14 days of expansion across all culture methods. Images are representative of all donors

To benchmark organoid expansion, we compared bioreactor performance to two established methods: traditional static droplet cultures and the current state-of-the-art spinner flasks. Total viable cell counts were measured at multiple time points across a two-week period (Fig. 2A; Suppl., Table S3). By day 14, the bioreactor system yielded an average of 5.63 × 10^8^ (± 1.1 × 10^8^) viable cells, compared to 1.22 × 10^8^ (± 4.26 × 10^7^) cells in spinner flasks and 4.02 × 10^5^ (± 2.81 × 10^5^) cells in static droplets (Fig. 2B). This represents a 1,400-fold increase in absolute yield compared to static cultures and nearly a 5-fold increase over spinner flasks, achieved with the same culture duration using a single culture unit. When normalized for seeding input, fold expansion was 375-fold in the bioreactor, 370-fold in spinner flasks, and 134-fold in static droplets, with the bioreactor showing a statistically significant advantage over static droplets (*p* = 0.022). While both dynamic systems demonstrated similar expansion efficiencies, only the bioreactor achieved high absolute yields in a single run, underscoring both scalability and reduced labor requirements.

Representative images of organoid morphology on days 2, 9, and 14 were selected to illustrate integrity and growth throughout the expansion phase. Across all culture formats, organoids maintained a consistent, translucent morphology characteristic of stemness throughout expansion (Fig. 2C), and showed comparable viability (Fig. 2D). Stemness was supported by LGR5 expression across modalities at the end of the 14-day expansion period (Fig. 2E). Importantly, there was no evidence of epithelial-to-mesenchymal transition; E-cadherin was comparably upregulated, while Vimentin – already low – remained stable in all conditions at the end of the expansion phase (Fig. 2E). Notably, no significant differences in these markers were observed between methods, indicating a robust, stable epithelial phenotype – also on a protein level (Fig. 2F). Additionally, liver organoids remained highly proliferative throughout the two-week expansion period, as indicated by the expression of Ki67 on both the gene-expression and protein levels (Fig. 2E/F).

### Integrated bioreactor workflow enables hepatic differentiation

**Fig. 3:**
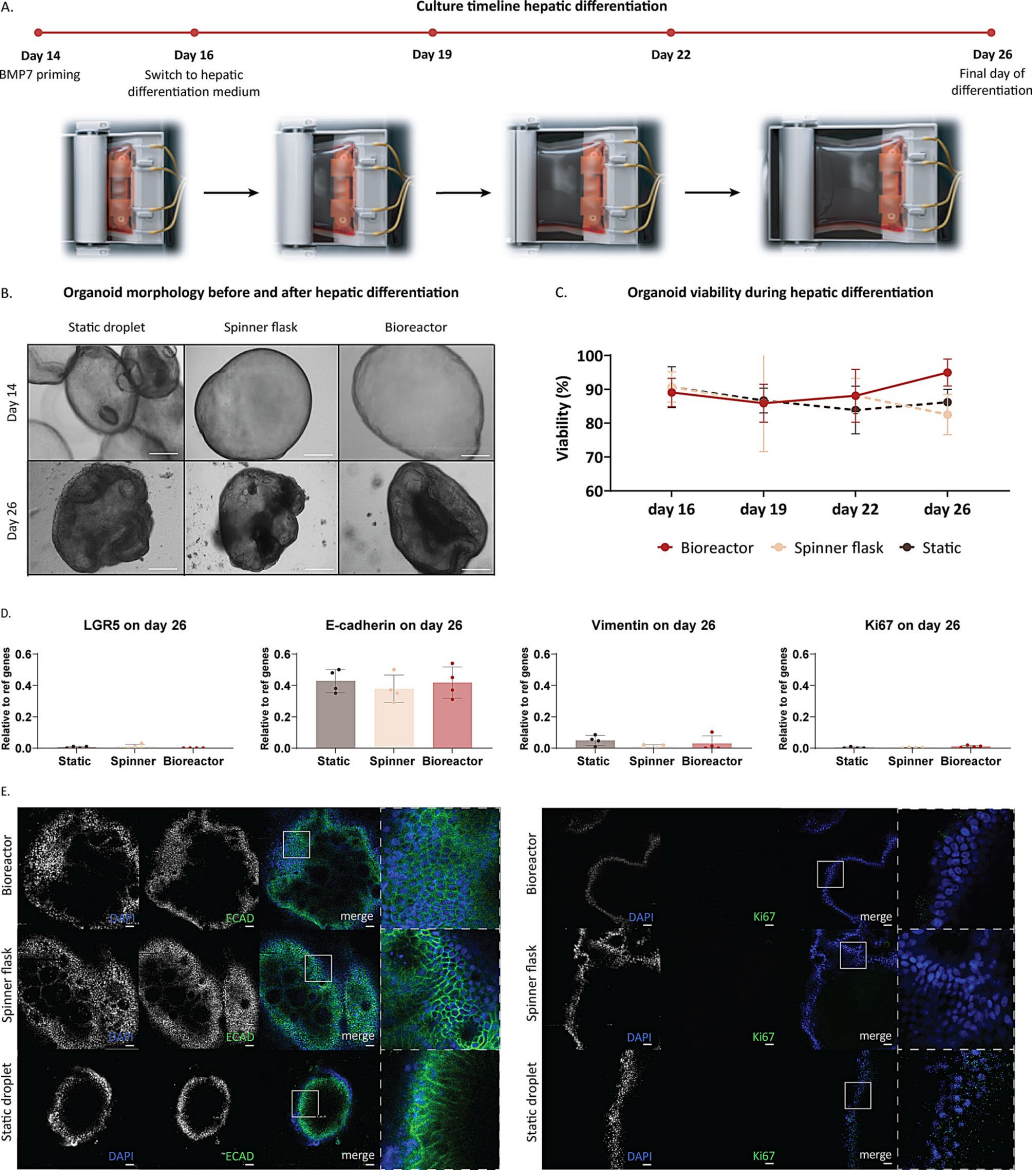
Integrated bioreactor workflow enables efficient hepatic differentiation. **A**. Schematic timeline of the hepatocyte differentiation culture protocol, starting with two days of BMP7 priming followed by 10 days of hepatocyte differentiation; **B**. Representative images show organoid morphology before and after hepatic differentiation, scale bar indicates 100 μm; **C**. Organoid viability over time for all modalities; **D**. Reduction of stemness marker LGR5, Vimentin, and Ki67 show successful differentiation, while maintaining the genetic expression of epithelial marker E-cadherin; **E**. Immunostaining for E-cadherin indicates that organoids retain an epithelial phenotype, while proliferation marker Ki67 is lost post-differentiation across all culture methods. Images are representative of all donors

**Fig. 4:**
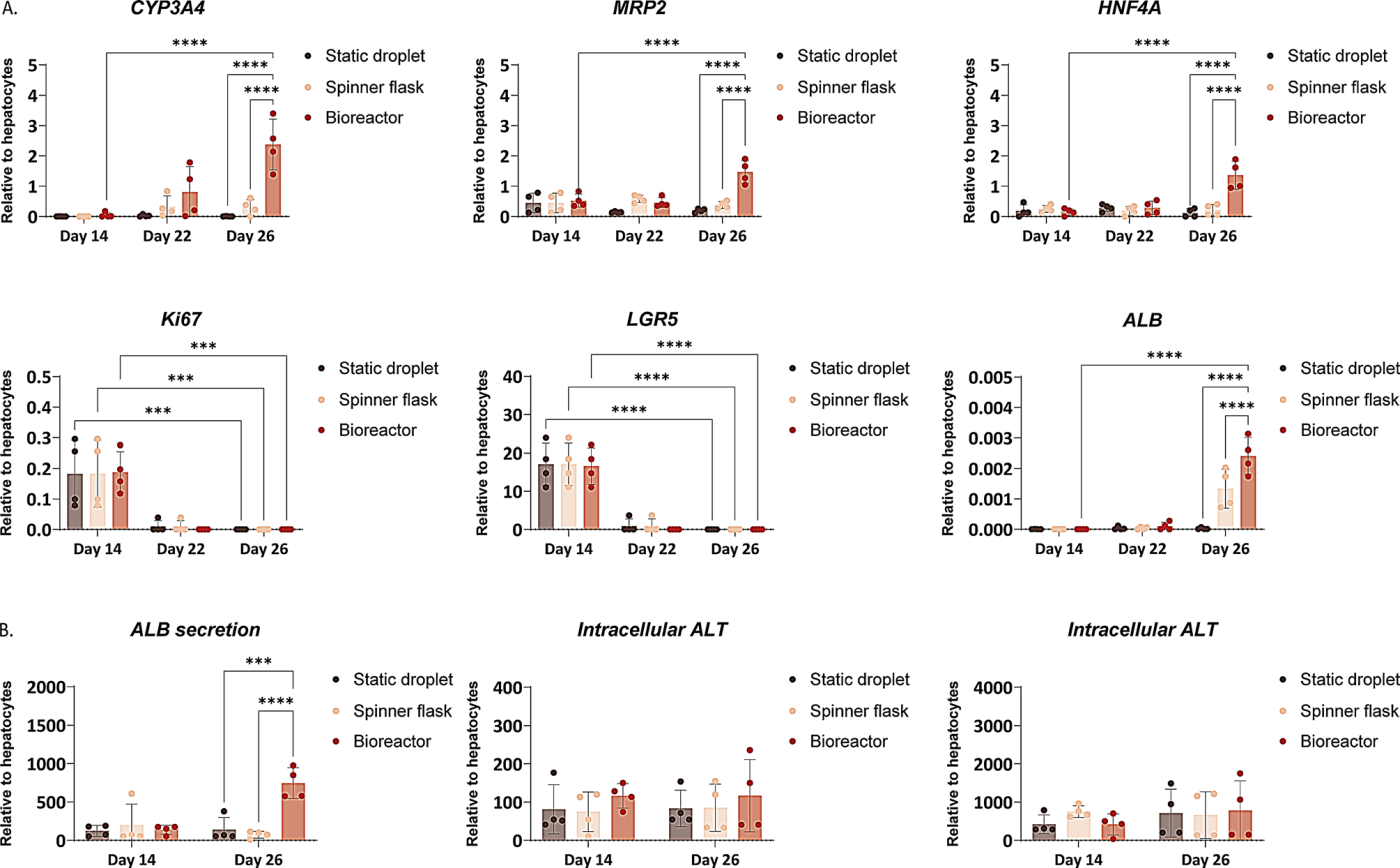
Bioreactor organoids exhibit enhanced hepatic gene expression and function. **A**. Gene expression profile confirms successful hepatocyte differentiation, as indicated by upregulation of hepatocyte markers CYP3A4, MRP2, HNF4A, and Albumin, and downregulation of Ki67 and LGR5; **B**. Albumin secretion is significantly higher in bioreactor organoids, while intracellular AST/ALT secretion remain comparable across the culture methods (corrected for cell numbers, mg/L/million and U/L/million)

Having established that liver organoids can be robustly expanded in the automated bioreactor, we next investigated whether they retain their capacity to differentiate into the hepatocyte lineage following large-scale expansion. To this end, we performed a proof-of-principle experiment in which organoids were transitioned directly from expansion to hepatocyte differentiation medium within the same closed-loop bioreactor system – without harvesting or subculturing. Mass-expanded organoids were first primed with BMP7 for two days, after which the medium was switched to hepatic differentiation conditions for an additional 10 days (Fig. 3A). Differentiation outcomes in the bioreactor were compared to those in spinner flasks and static droplets. As expected, organoids became darker and more compact during hepatic differentiation (Fig. 3B), while remaining highly viable (Fig. 3C). Differentiated organoids retained epithelial integrity following hepatic differentiation, as confirmed by absence of Vimentin expression and sustained E-cadherin expression on a transcript (Fig. 3D) and protein level (Fig. 3E). In all culture formats, proliferative activity decreased, as indicated by the loss of Ki67 expression at both transcripts (*p* = 0.0051, Fig. 3D) and protein level (Fig. 3E). Furthermore, gene expression analysis confirmed lineage specification. *LGR5* expression was significantly downregulated (*p* < 0.0001; Fig. 3D), whereas hepatic markers including *HNF4A*, *CYP3A4*, *MRP2*, and *ALB* were significantly upregulated in the bioreactor compared to the expansion phase, demonstrating retention of hepatic differentiation capacity (*p* < 0.0001; Fig. 4A). At the end of differentiation (day 26), expression of *CYP3A4*, *MRP2*, *HNF4A*, and *ALB* was also significantly higher in bioreactor-derived organoids than those differentiated in static droplets and spinner flasks (*p* < 0.0001). Functional readouts further demonstrated liver-specific activity. Following differentiation, organoids exhibited a significant increase in Albumin secretion exclusively in bioreactor cultures (*p* < 0.0001), while intracellular AST and ALT levels remained comparable across all culture methods (Fig. 4B), indicating maintenance of metabolic activity without signs of cellular damage.

### Dynamic shifts in metabolism and oxygen consumption accompany large-scale liver organoid expansion

While donor-to-donor variation in organoid expansion did not reach statistical significance, consistent trends in metabolic activity were observed across donors. To ensure meaningful cross-comparison, a standardized culture configuration (“design-freeze”) was adopted across all donors, maintaining identical seeding densities, medium formulations, and process parameters. We monitored key metabolic parameters – glucose, lactate, and DO_2_ – during the automated bioreactor culture to relate metabolic dynamics to cell yield. This controlled setup enabled assessment of how metabolic activity evolves during high-density expansion and served as an initial exploration of potential critical process parameters (CPPs) for robust organoid growth.

**Fig. 5:**
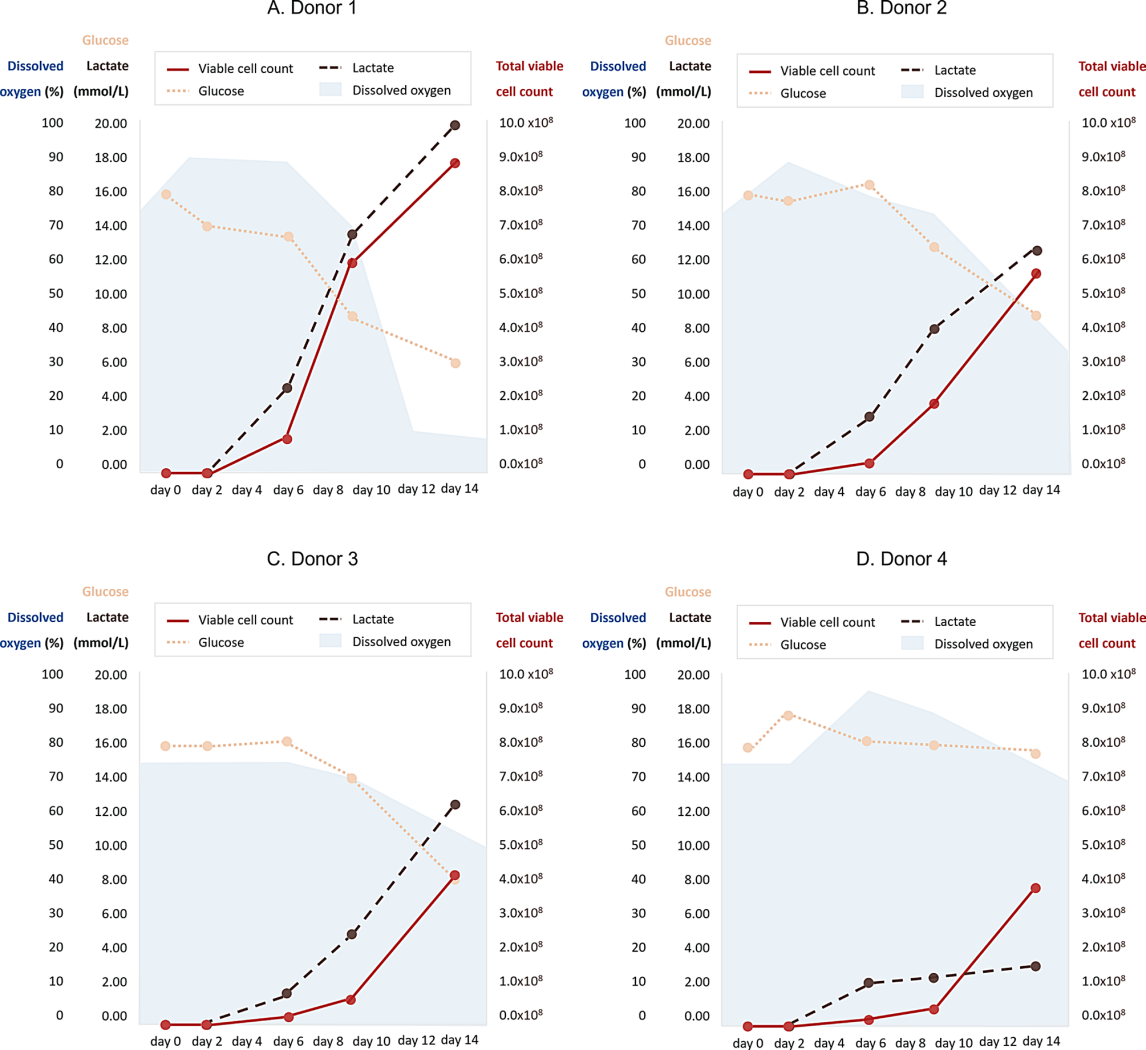
Trend between metabolism, oxygen, and cell counts for all donors. **A**-**D**. Trend graphs for donors 1–4 showing glucose, lactate and dissolved oxygen (DO_2_) levels plotted against total cell yield over time in the automated cultures. Glucose consumption and lactate production are shown to correlate with cell proliferation, while DO_2_ levels decline as cell density increases, reflecting increased metabolic demand

As shown in Fig. 5, all donors followed a similar metabolic trajectory: as cell numbers increased, gradual glucose consumption intensified, accompanied by lactate accumulation and increased oxygen utilization. These features reflect the characteristic high glycolytic activity combined with elevated pyruvate oxidation that supports rapid proliferation of ICOs, rather than donor-specific metabolic differences. Cultures with steeper growth curves displayed earlier and more pronounced glucose depletion and oxygen consumption, suggesting that metabolic demand scales with proliferation rate. During peak expansion (> 4 × 10⁸ cells), oxygen consumption occasionally challenged the system’s ability to maintain the 75% DO_2_ setpoint, with one donor showing transient drops to as low as 10% DO_2_.

Importantly, these fluctuations did not compromise organoid viability, phenotype or function, demonstrating that ICOs can adapt to fluctuating oxygen levels during intense growth phases. Together, these findings emphasize the need to optimize metabolic support – particularly glucose and oxygen availability – to sustain high expansion rates. Rather than donor-tailored control, fine-tuning these parameters may serve as a generalizable strategy to ensure consistent and reproducible organoid yields across donor sources.

### Scaling up using bioreactors is more cost-effective than static cultures

Beyond total cell yield, operational burden and material use are critical factors in evaluating the clinical feasibility of cell culture methods. To gain insight in these factors, we compared the automated bioreactor, spinner flask, and static droplet cultures in terms of manual labor and material usage (Table 1).

**Table 1:** Cost-effectiveness calculations for expansion protocols

	Bioreactor	Spinner flask	Static droplet
Average uncorrected yield	5.63 × 10^8^ cells	1.22 × 10^8^ cells	4.02 × 10^5^ cells
Units required	0.71 culture bag	1 spinner flask	1 well 12 wp
Total volume	850 mL	150 mL	1.1 mL
Required medium volume	765 mL (95%)	135 mL (90%)	1.0 mL
Required Matrigel volume	42.5 mL (5%)	15 mL (10%)	0.1 mL
Expected average yield	5.63 × 10^8^ cells	5.63 × 10^8^ cells	5.63 × 10^8^ cells
Units required	0.71 culture bag	4.6 spinner flasks	1,400 wells 12 wp
Total volume	850 mL	690 mL	1,540 mL
Required medium volume	765 mL (95%)	621 mL (90%)	1,400 mL
Required Matrigel volume	42.5 mL (5%)	69 mL (10%)	140 mL
Medium usage compared to bioreactor (fold)		0.81	1.83
Matrigel usage compared to bioreactor (fold)		1.62	3.29

Comparison of material usage and culture efficiency across bioreactor, spinner flask, and static droplet protocols. The upper section shows experimentally obtained yields and corresponding culture volumes. The lower section normalizes all methods to an equivalent yield to enable direct comparison of material requirements. Fold differences relative to the bioreactor are summarized. The relative estimates are based on our standardized assumptions for materials and labor

In the bioreactor, the average yield of 563 million cells was achieved using only ~ 70% of the culture bag’s volume capacity. In contrast, a single spinner flask yielded approximately 122 million cells, meaning ~ 5 spinner flasks are needed to match the automated bioreactor’s potential output. For static cultures, one well of a 12-well plate yields on average 400,000 cells, implying that nearly 1,400 wells, or around 120 twelve-well plates, would be needed to reach an equivalent cell yield. With regards to labor, the automated bioreactors total volume can be easily refreshed or harvested by a single operator within 10 min, whereas refreshing and harvesting more than 120 plates would require several hours for multiple trained operators, or expensive automated plate-handling systems. In addition to an improvement in labor- and time-savings, the automated bioreactor also demonstrated greater efficiency. Static droplets required approximately 3.3 times more Matrigel and 1.8 times more culture medium, making the bioreactor approach significantly more cost effective in terms of material costs. In a GMP setting, a single closed bioreactor run enables high-yield production with fewer open handling steps, reducing labor and contamination risk during seeding, feeding, and harvesting. Compared with static plates and spinner flasks, which require parallel processing, this simplified workflow approach also reduces cleanroom footprint and GMP documentation complexity.

## Discussion

The urgent need for viable alternatives to the limited availability and frequent unsuitability of donor livers has fueled the development of organoid-based technologies for regenerative medicine [31, 32]. ICOs, with their unique bipotential characteristics, offer a promising solution to bridge this gap. However, culturing ICOs in conventional static cultures or more advanced agitation-based platforms like spinner flasks falls short of meeting the demand for the billions (10^9^) of cells required for potential therapeutic applications or large in vitro screens. This scale-up gap is increasingly recognized, with growing emphasis on automated, sensor-controlled culture platforms that may enable more standardized and scalable organoid manufacturing for drug development and regenerative medicine [22, 33, 34].

For hepatocyte cell therapy, approximately 1–2 × 10⁸ cells per kilogram of body weight are required [12, 32], meaning up to 1–2 × 10¹⁰ cells may be needed for an adult patient. At our current day-14 yields, a single bioreactor run produces 8.0 × 10⁸ cells, equating to roughly 13–25 runs per dose. Although substantial, automated bioreactor runs are far more feasible than managing hundreds of spinner flasks or thousands of well plates, supporting the practicality of this approach for clinical-scale manufacturing. Moreover, hepatocyte transplantation is typically administered over multiple infusions [32, 35], which further supports the feasibility of bioreactor-based production for clinical applications. Importantly, bioreactor-expanded ICOs retained efficient hepatic differentiation capacity within the same closed system, minimizing handling and contamination risk. Although absolute albumin secretion remained below in vivo levels, the observed hepatocyte-marker upregulation further points towards a retained hepatic differentiation potential. Systematic functional characterization and longer-term assessment of phenotype stability will be required before therapeutic application can be considered. The higher albumin output observed after bioreactor differentiation may reflect more homogeneous oxygen and nutrient availability during differentiation, as controlled mixing and oxygen transfer can reduce gradients that otherwise limit metabolic maturation in static formats. At the same time, hydrodynamic forces associated with mixing (e.g., shear stresses) are likely to influence organoid integrity and growth during scale-up, although these were not quantitatively characterized here. Internal CFD analyses performed previously for a microcarrier-based application using the same platform indicated substantially lower energy dissipation rates than a comparable stirred-tank configuration. However, these results are not directly transferable to organoid culture due to differences in geometry, rheology, and 3D structure properties. In the present study, the preserved morphology, high viability, maintained epithelial identity, and hepatic differentiation capacity under these conditions suggest that the optimized agitation regime did not impose overtly damaging forces. Quantitative hydrodynamic characterization will be addressed in future optimization studies (e.g., via computational fluid dynamics or flow characterization), which will be important to support process qualification and clinical translation. Furthermore, the same closed-system workflow could also support cholangiocyte differentiation, enabling bile-duct repopulation, which requires substantially fewer cells [16].

A limitation of the present study is that we did not directly assess genomic stability, epigenetic state, phenotypic drift, or culture-induced selection pressures during large-scale expansion. Although related studies using the same organoids have reported genomic integrity over passages and included in vivo safety assessments [4, 15, 16, 23], these endpoints were not evaluated here and remain essential for future qualification of this process for clinical use. Dedicated longitudinal studies assessing genomic stability, epigenetic changes, and culture-induced alterations will therefore be required before clinical implementation.

Persistent bottlenecks in hepatobiliary organoid translation include incomplete maturation, limited microenvironmental complexity, and insufficient process standardization [36, 37]. In this context, the present study shows that a closed, automated bioreactor enables controlled and scalable ICO expansion. Because static and spinner culture conditions were not exhaustively optimized across all possible configurations, the comparative findings should be interpreted within the conditions tested here, under which the bioreactor provided advantages in scale, handling, and process control. Beyond yield, monitoring of glucose, lactate, and DO₂ provided valuable insight into metabolic and proliferative behavior, and may support more standardized refreshment and control strategies to enable early prediction of success and demonstrate donor-to-donor differences. Although the present data support feasibility across the donor set tested here, broader validation across additional donors, including clinically relevant donor types, will be required to define biological variability and support translational implementation. Donors showing steeper expansion rates are expected to exhibit higher oxygen consumption, reflected by faster DO_2_ decreases and/or higher oxygen supplementation requirements, and a steeper decrease in glucose and increase in lactate. This supports defining critical process parameters (CPPs) such as DO_2_ setpoints during expansion and differentiation, as well as metabolite-based control limits (e.g., glucose floor and lactate ceiling) that can be tuned per donor to optimize yield while preserving phenotype and differentiation capacity. Our work directly addresses the manufacturing component of the posted challenges by demonstrating a closed, monitored workflow and by enabling in-process readouts that can support better standardization and donor-responsive bioprocessing – a concept increasingly relevant for personalized cell therapy and precision tissue engineering [38–40]. However, achieving higher functional maturation and more physiological complexity will likely require integration with defined matrices and/or multicellular cocultures.

From a GMP-alignment perspective, the use of closed-system operation and single-use culture bags supports a reduced-contamination risk workflow and can facilitate standardized manufacturing with fewer open handling steps. In addition, real-time monitoring (e.g., DO_2_ and metabolite trends) can support predefined in-process controls and batch-release comparability – features that are typically expected in GMP-oriented process development. While this study addresses key translational bottlenecks, several critical steps remain before clinical application is feasible. Large‑scale expansion must be paired with systematic stability assessments, encompassing genomic integrity, epigenetic drift, and culture‑induced transcriptional or functional changes across passages and production runs, to ensure safety and reproducibility [41–43]. Prolonged in vitro culture can introduce selection pressures that influence safety, potency, and reproducibility. In parallel, standardized quality‑control and release criteria will be essential, encompassing identity, purity, potency, and safety assays aligned with regulatory expectations [44, 45]. Full clinical translation will also require replacing non‑defined components such as Matrigel, due to their undefined composition, and batch-to-batch variability, with xeno‑free, chemically defined alternatives, such as Invasin-functionalized PIC hydrogels, and validating product comparability under GMP‑compatible conditions [46]. Collectively, these considerations position the present work as an important first step toward clinically scalable organoid manufacturing. Further optimization of growth conditions and handling will be essential for full GMP compliance, and batch consistency, run-to-run reproducibility, economic modelling, and regulatory-aligned validation remain to be established in future studies.

Importantly, while manufacturing capability is a prerequisite for translation, therapeutic applicability ultimately requires demonstration of in vivo safety and efficacy. The primary focus of this study was to address the upstream manufacturing bottleneck (establishing an automated, closed, and monitored workflow that expands human liver organoids to clinically relevant cell numbers while preserving phenotype and differentiation capacity) rather than to evaluate in vivo functional rescue. Future work should therefore pair this manufacturing platform with dedicated transplantation studies to assess biodistribution, engraftment efficiency, and functional benefit in appropriate disease models. As engraftment efficiency continues to be a major hurdle to clinical application, it remains to be determined whether organoids should be implanted at the expansion or differentiation stage, an important consideration for optimizing therapeutic efficacy. To ensure clinical readiness, future development should also include standardized quality control measures, safety and functionality assays, and alignment with regulatory requirements. The cost calculations presented in this study were limited to material use and culture labor under non-GMP research conditions; cleanroom operation and compliance costs were not included but would substantially increase total expenses across all methods.

## Conclusion

Building on these findings, this work represents an early but significant step toward standardized, GMP-adaptable liver organoid manufacturing. While the current work establishes a proof-of-principle for large-scale hepatic differentiation, further studies are needed for in vivo validation, including engraftment, tissue integration, and functional recovery. In the long term, scalable bioreactor technologies may underpin GMP-ready workflows that support not only therapeutic implementation but also organoid-based disease modeling and drug discovery, advancing the field toward reproducible, patient-ready alternatives to donor organs.

## Electronic Supplementary Material

Below is the link to the electronic supplementary material.

Supplementary Material 1

## Data Availability

All data generated or analyzed during the current study are available from the lead contact (Bart Spee (b.spee@uu.nl)) on reasonable request. Organoids were derived from healthy donor tissue and cannot be publicly shared due to privacy and consent restrictions, but metadata and experimental outputs necessary to interpret and reproduce the findings can be provided upon request. All commercial reagents and homemade media formulations used in this study are described in the Methods section and additional details can be obtained from the corresponding author.

## Abbreviations

ALB: Albumin
ALT: Alanine aminotransferase
AST: Aspartate aminotransferase
BMP7: Bone morphogenetic protein 7
cDNA: Complementary DNA
CPPs: Critical process parameters
CYP3A4: Cytochrome P450 family 3 subfamily A member 4
DMEM: Dulbecco’s Modified Eagle Medium
DO_2_: Dissolved oxygen
ECM: Extracellular matrix
EM: Expansion medium
GMP: Good Manufacturing Practice
HDM: Hepatic differentiation medium
HNF4A: Hepatocyte nuclear factor 4 alpha
HOs: Hepatic organoids
HPRT: Hypoxanthine-guanine phosphoribosyltransferase
ICOs: Intrahepatic cholangiocyte organoids
LGR5: Leucine-rich repeat-containing G-protein coupled receptor 5
MRP2: Multidrug resistance-associated protein 2
OWB: Organoid washing buffer
RPL19: Ribosomal protein L19
YWHAZ: Tyrosine 3-monooxygenase/tryptophan 5-monooxygenase activation protein zeta
