# Supplementary material for: Automated and scalable expansion of human liver organoids for translational applications
Source: J Transl Med. 2026 Apr 24;24:754. doi: 10.1186/s12967-026-08169-z (PMC13255226; doi:10.1186/s12967-026-08169-z)
Supplement: Supplementary file 1 — Supplementary Material 1 [file 12967_2026_8169_MOESM1_ESM.docx]

**Supplemental Information**

**Liver organoid donors**

Four donors were used for all experiments, unless specified otherwise. Donor information regarding sex, age, passage number, and cause of death are depicted in the table below.

**Table S1 – Organoid donor information**

|  | **Donor 1** | **Donor 2** | **Donor 3** | **Donor 4** |
| --- | --- | --- | --- | --- |
| **Donor sex** | Female | Female | Male | Male |
| **Donor age** | 49 years | 24 years | 51 years | 70 years |
| **Passage** | 7 | 7 | 9 | 8 |
| **Donor type** | DBD | DCD | DBD | DBD |

**Optimized parameter settings for the automated bioreactor**

Parameter settings were optimized to suit the large-scale expansion of liver organoids.

**Table S2 – Automated bioreactor parameter settings**

| **Parameter** | **Dimension** | **Setting** | **Description** |
| --- | --- | --- | --- |
| **Max angle** | Degrees (°) | 180 | Max. rotation angle along its longitudinal axis |
| **Speed** | (°/second) | 250 | Speed of rotation along its longitudinal axis |
| **Acceleration** | (°/second^2^) | 250 | Acceleration until designated speed |
| **Deceleration** | (°/second^2^) | 250 | Deceleration until speed is 0°/s |
| **Vertical pause** | (time, e.g. sec) | 0 | Vertical hold time at maximum angle |
| **Horizontal pause** | (time e.g. hours) | 0 | Horizontal hold time at rest position |

**Cell counts per modality per donor**

Cell expansion was measured using the cell samples taken over time. Viable cell counts measured over time per modality per donor show expansion over time and can be viewed in Table S3.

**Table S3 – (Mean) viable cell counts over time per modality per donor**

|  |  | Bioreactor | Spinner flask | Static droplet |
| --- | --- | --- | --- | --- |
| Donor 1 | Day 0 | 1.50x10^6^ | 3.30x10^5^ | 3.00x10^3^ |
|  | Day 2 | 5.56 x10^6^ | 2.48x10^6^ | 1.12x10^3^ |
|  | Day 6 | 7.64x10^7^ | 1.68x10^7^ | 1.25x10^4^ |
|  | Day 9 | 6.02x10^8^ | 5.81x10^7^ | 5.54x10^4^ |
|  | Day 14 | 8.82x10^8^ | 2.40x10^8^ | 1.24x10^6^ |
| Donor 2 | Day 0 | 1.50x10^6^ | 3.30x10^5^ | 3.00x10^3^ |
|  | Day 2 | 8.82x10^6^ | 4.05x10^5^ | 6.60x10^3^ |
|  | Day 6 | 2.08x10^7^ | 9.62x10^5^ | 1.93x10^4^ |
|  | Day 9 | 1.86x10^8^ | 2.92x10^6^ | 4.58x10^4^ |
|  | Day 14 | 5.65x10^8^ | 3.83x10^7^ | 1.55x10^5^ |
| Donor 3 | Day 0 | 1.50x10^6^ | 3.30x10^5^ | 3.00x10^3^ |
|  | Day 2 | 2.26x10^6^ | 3.84x10^6^ | 3.07x10^3^ |
|  | Day 6 | 1.12x10^7^ | 4.44x10^6^ | 1.30x10^4^ |
|  | Day 9 | 4.84x10^7^ | 1.87x10^7^ | 6.42x10^4^ |
|  | Day 14 | 4.15x10^8^ | 1.18x10^8^ | 2.00x10^5^ |
| Donor 4 | Day 0 | 1.50x10^6^ | 3.30x10^5^ | 3.00x10^3^ |
|  | Day 2 | 1.90x10^6^ | 7.13x10^5^ | 2.24x10^3^ |
|  | Day 6 | 1.57x10^7^ | 3.00x10^6^ | 4.01x10^3^ |
|  | Day 9 | 3.64x10^7^ | 5.74x10^6^ | 1.07x10^4^ |
|  | Day 14 | 3.91x10^8^ | 9.35x10^7^ | 1.98x10^4^ |
| Average | Day 0 | **1.50x10^6^** | **3.30x10^5^** | **3.00x10^3^** |
|  | Day 2 | **4.64x10^6^** | **1.86x10^6^** | **3.26x10^3^** |
|  | Day 6 | **3.10x10^7^** | **6.29x10^6^** | **1.22x10^4^** |
|  | Day 9 | **2.18x10^8^** | **2.14x10^7^** | **4.40x10^4^** |
|  | Day 14 | **5.63x10^8^** | **1.22x10^8^** | **4.02x10^5^** |

**Culture medium**

The same culture medium was used in all culture methods. Culture medium during the expansion phase (expansion medium; EM) can be found in Table S4, and medium during the differentiation phase (hepatic differentiation medium; HDM) can be found in Table S5.

**Table S4 – Expansion medium (EM)**

|  | **Concentration** | **Company details** |
| --- | --- | --- |
| Advanced DMEM/F12 | N.A. | Gibco 12634010 |
| Penicilin / streptomycin | 1% v/v | Gibco 15140122 |
| GlutaMax | 1% v/v | Gibco 35050061 |
| HEPES | 10 mM | Gibco 15630080 |
| RSPO3-FC Fusion protein medium | 10% v/v | Immunoprecise Antibodies, R001 |
| B27 supplement without vitamin A | 2% v/v | Invitrogen, 12587010 |
| N2 supplement | 1% v/v | Invitrogen, 17502001 |
| Nicotinamide | 10 mM | Sigma-Aldrich, N0636 |
| N-acetylcysteine | 1.25 mM | Sigma-Aldrich, A9165 |
| Fibroblast growth factor 10 | 100 ng/mL | Peprotech,100-26 |
| Recombinant human (Leu15)-gastrin 1 | 10 nM | AnaSpec, AS-64149 |
| Forskolin | 10 µM | Sigma-Aldrich, F3917 |
| Epidermal growth factor | 50 ng/mL | Miltenyi biotec, 130-093-825 |
| Hepatocyte growth factor | 25 ng/mL | Miltenyi biotec, 130-103-437 |
| A83-01 | 5 µM | Stemcell Technologies, 100-0245 |
| Primocin | 50 µg/mL | Invivogen, ant-pm-05 |

**Table S5 – Hepatic differentiation medium (HDM)**

|  | **Concentration** | **Company details** |
| --- | --- | --- |
| Advanced DMEM/F12 | N.A. | Gibco 12634010 |
| Penicilin / streptomycin | 1% v/v | Gibco 15140122 |
| GlutaMax | 1% v/v | Gibco 35050061 |
| HEPES | 10 mM | Gibco, 15630080 |
| B27 supplement without vitamin A | 2% v/v | Invitrogen, 12587010 |
| N2 supplement | 1% v/v | Invitrogen, 17502001 |
| Dexamethasone | 30 µM | Sigma-Aldrich, D4902 |
| N-acetylcysteine | 1.25 mM | Sigma-Aldrich, A9165 |
| Fibroblast growth factor 19 | 100 ng/mL | Abcam, ab283418 |
| Recombinant human (Leu15)-gastrin 1 | 10 nM | AnaSpec, AS-64149 |
| DAPT | 10 µM | Selleck Chemicals, S2215 |
| Epidermal growth factor | 50 ng/mL | Miltenyi biotec, 130-093-825 |
| Hepatocyte growth factor | 25 ng/mL | Miltenyi biotec, 130-103-437 |
| A83-01 | 5 µM | Stemcell Technologies, 100-0245 |
| Human BMP7 recombinant protein | 25 ng/mL | Miltenyi biotec, 130-103-436 |
| Primocin | 50 µg/mL | Invivogen, ant-pm-05 |

**Refreshment regimes and continuous expansion in the bioreactor**

For both suspension cultures, refreshment regimes included the supplementation of fresh medium including Matrigel to the existing cell suspension on day 2, 6, 9, 12 and 14. In Table S6 the refreshment regimes and sample volumes taken at different timepoints can be found for spinner flask and bioreactor cultures.

**Table S6 – Refreshment regimes for suspension cultures**

|  | **Spinner flasks** | | **Bioreactor** | |
| --- | --- | --- | --- | --- |
|  | *Medium* (incl. 5% Matrigel) | *Sample* | *Medium* (incl. 5% Matrigel) | *Sample* |
| Day 0 | 22 mL | - | 100 mL | - |
| Day 2 | 40 mL | -4 mL | 190 mL | -10 mL |
| Day 6 | 72 mL | -4 mL | 380 mL | -10 mL |
| Day 9 | 108 mL | -4 mL | 620 mL | -10 mL |
| Day 12 | 134 mL | -4 mL | 810 mL | -10 mL |
| Day 14 | 130 mL | -4 mL | 800 mL | -10 mL |

Tube welders and sealers were required for refreshing bioreactor cultures. At each time point the roller-set-point value, connected to the roller position on the rocker platform, was increased to accommodate the additional volume of fresh culture medium (Fig. 2A). During this process, the suspension bag is rolled out, increasing the capacity of the bag. The weldable tubing from the bottom compartment of the suspension bag was connected to the weldable tubing of the vessel containing fresh medium. Fresh medium was gently transferred into the bag. The vessel was then detached with a tube sealer, thereby completing the closed loop refreshing process, and the suspension bag was rolled bag to the newly set medium volume. This process was repeated at the point of differentiation to switch out EM for HDM without removing the cultured organoids. Due to the presence of the filter in the suspension bag, the culture medium could be drained into a waste bag, the cells were washed with basal DMEM, and the cultures were replenished with fresh HDM (incl. 5% Matrigel).

**Quantitative PCR**

Quantitative PCR was performed to assess stem cell, proliferation, and hepatic differentiation capacity after large-scale expansion and hepatic differentiation. Primers used for this analysis can be found in Table S7.

**Table S7 – Details of selected qPCR primers**

| **Protein** | **Forward primer** | **Reverse primer** | **Temp (°C)** | **Size** |
| --- | --- | --- | --- | --- |
| LGR5 | GCAGTGTTCACCTTCCC | GGTCCACACTCCAATTCTG | 64 | 68 |
| Ki67 | GCTACTCCAAAGAAGCCTGTG | AAGTTGTTGAGCACTCTGTAGG | 60 | 95 |
| Ecadherin | AGGCCAAGCAGCAGTACATT | ATTCACATCCAGCACATCCA | 60 | 110 |
| Vimentin | ACACCCTGCAATCTTTCAGACA | GATTCCACTTTGCGTTCAAGGT | 60 | 76 |
| Albumin | GTTCGTTACACCAAGAAAGTACC | GACCACGGATAGATAGTCTTCTG | 64 | 110 |
| CYP3A4 | TTTTGTCCTACCATAAGGGCTTT | CACAGGCTGTTGACCATCAT | 62.5 | 95 |
| HNF4a | GTACTCCTGCAGATTTAGCC | CTGTCCTCATAGCTTGACCT | 58 | 162 |
| MRP2 | GGGATCTCTTCCACACTGGAT | CATACAGGCCCTGAAGAGGA | 64 | 91 |
| KRT19 | CTTCCGAACCAAGTTTGAGAC | AGCGTACTGATTTCCTCCTC | 64 | 183 |
| SLC4A2 | ACTACCTGAGTGACTTCCGA | TGCTACAGAACGAGAAGAAGG | 64 | 266 |

**Wholemount immunofluorescence**

Wholemount immunofluorescence was used to assess E-cadherin and Ki67 expression after large-scale expansion and hepatic differentiation. Antibodies used for this analysis can be found in Table S8.

**Table S8 – Details of primary and secondary antibodies**

|  | **Host** | **Dilution** | **Company details** |
| --- | --- | --- | --- |
| E-cadherin | Mouse | 1:50 | BD Biosciences, 610181 |
| Ki67 | Mouse | 1:100 | Dako, M7240 |
| Goat anti-Mouse IgG Alexa 488 | Goat | 1:1000 | ThermoFisher Scientific, A-11029 |
